# Risk factors for systemic and venous thromboembolism, mortality and bleeding risks in 1125 patients with COVID-19: relationship with anticoagulation status

**DOI:** 10.18632/aging.202769

**Published:** 2021-03-26

**Authors:** Wencheng Li, Zhifeng Xu, Huiling Xiang, Chun Zhang, Yutao Guo, Jing Xiong

**Affiliations:** 1 Department of Urology, Union Hospital, Tongji Medical College, Huazhong University of Science and Technology, Wuhan 430022, China; 2 Department of Nephrology, Union Hospital, Tongji Medical College, Huazhong University of Science and Technology, Wuhan 430022, China; 3 Medical School of Chinese PLA, Department of Cardiology, Chinese PLA General Hospital, Beijing 100853, China; 4 Liverpool Centre for Cardiovascular Sciences, University of Liverpool and Liverpool Heart and Chest Hospital, Liverpool L69 3BX, United Kingdom

**Keywords:** COVID-19, thromboembolism, bleeding, death

## Abstract

Aim: Coronavirus disease 2019 (COVID-19) has been associated with increased mortality and morbidity from thromboembolism, especially venous thromboembolism. There are more limited data for systemic thromboembolism. The present study aimed to investigate the prevalence of systemic and venous thromboembolism as well as major bleeding and mortality in relation to underlying risk factors and the impact of anticoagulation use in hospitalized patients with COVID-19.

Methods and results: Patients with COVID-19 admitted to Union Hospital, Wuhan, Hubei, China between January 08, 2020 and April 7, 2020 were enrolled in this retrospective study. Cox proportional hazard models were utilized to determine associated risk factors for clinical events, adjusting for the severity of COVID-19 infection, drug therapies, comorbidities, surgery, and use of antithrombotic drugs.

There were 1125 patients (49.9% male; mean age 58 years (standard deviation, SD, 15 years)) with a mean follow-up of 21 (SD 13) days. Approximately 25 (30%) patients with thromboembolism also suffered bleeding events.

Age was an independent risk factor for thromboembolism, bleeding events, and death (all p<0.05). After adjusting for the severity of COVID-19 infection, comorbidities, surgery, antiviral drugs, immunomodulators, Chinese herbs, and antithrombotic drugs, low lymphocyte counts (hazard ratio, HR, 95% confidence interval (CI), 1.03, 1.01-1.05, p=0.01) and surgery (HR 2.80, 1.08-7.29, p=0.03) independently predicted the risk for major bleeding, whereas liver dysfunction (HR 4.13, 1.30-13.1, p=0.02) was an independent risk factor for patients with both thromboembolism and bleeding events.

Conclusions: Patients with COVID-19 were at high risk for thromboembolic and bleeding events as well as mortality. The use of anticoagulants, especially parenteral anticoagulants, significantly reduced the risk for composite outcomes of thromboembolism, bleeding events, and death. The presence of AF was a contributor to systemic thromboembolism in COVID-19 patients.

## INTRODUCTION

The coronavirus disease 2019 (COVID-19) pandemic has resulted in approximately 3 million infections globally, with over 200,000 deaths, mainly due to severe acute respiratory syndrome and multiorgan dysfunction [1]. In studies conducted during the early stage of the COVID-19 outbreak, various hemostatic abnormalities were reported, including thrombocytopenia, prolongation of prothrombin time, international normalized ratio and thrombin time, elevated D-dimer levels, etc. [2–6]. Increased mortality and morbidity from thromboembolism, especially venous thromboembolism, have been reported, commonly in critically ill COVID-19 patients [7–9]. These acute thromboembolic events were strongly associated with worsening outcomes, highlighting the necessity for thromboembolism risk management [10].

Despite the data on venous thromboembolism in COVID-19, there are more limited data on systemic thromboembolism and the value of anticoagulation regimens in balancing the risks of major bleeding. One study showed that anticoagulants reduced mortality in COVID-19 patients with D-dimer > 3.0 μg/mL [11], but there appear to be empiric therapeutic anticoagulation approaches in current practice. The appropriate choice of anticoagulant type, together with the appropriate dosages for thromboprophylaxis, remains unclear in the absence of published randomized trials. For example, intermediate-dosage low-molecular-weight heparin (LMWH) thromboprophylaxis (40-60 mg) is commonly used in Chinese patients [11], whereas higher dosages (i.e., 80-100 mg) are considered in Italy [12].

High thromboembolism risks have been reported in severely or critically ill patients, but there are fewer data in relation to underlying risk factors and the impact of anticoagulation use in patients with COVID-19 on systemic and venous thromboembolism separately as well as on major bleeding and mortality. Indeed, there are complex interactive factors that could impact both thromboembolic and bleeding risks that would influence decision making, including the severity of COVID-19 infection, comorbidities, surgery or interventional procedures, drug therapies, and the use of antithrombotic drugs.

In the present study, our aim was to investigate the prevalence of systemic and venous thromboembolism as well as major bleeding and mortality in relation to underlying risk factors and the impact of anticoagulation use among hospitalized patients with COVID-19.

## RESULTS

We included 1,125 patients (561, 49.9% male; mean age 58 (SD 15) years, range 14-97 years) with a mean follow-up of 21 (SD 13) days (Table 1). Of them, 408 (36.3%) were aged over 65 years, and 33 (2.9%) underwent surgery; admission specialties are summarized in Supplementary Table 1. The average duration of symptoms to hospital admission was 17 days (SD 11). Hypertension, diabetes mellitus, and coronary artery disease were the most common comorbidities in this population (Table 1). Antiviral drugs were used by the admitting team in 788 (70.0%) patients, and glucocorticoid drugs were used in 203 (18.0%) patients. Anticoagulants were used in 249 (22.1%) patients: 87 (7.7%) oral anticoagulants, 209 (18.6%) parenteral anticoagulants (intermediate dose LMWH, or intravenous heparin), and 47 (4.2%) were prescribed both oral anticoagulants plus parenteral anticoagulants (Table 2).

**Table 1:** Baseline characteristics of 1125 patients with COVID-19.

	**Patients (n=1125)**
**Age, mean, SD**	58.3 (15.1)
Age ≤ 17	3 (0.3)
Age 18-64	714 (63.5)
Age 65-74	268 (23.8)
Age 75-84	102 (9.1)
Age ≥ 85	38 (3.4)
**Male, n (%)**	561 (49.9)
**Days to admission, mean, SD**	17.1 (11.5)
**Respiratory rate** (time/minute), mean, SD	22 (4)
**Heart rate** (time/minute), mean, SD)	91 (27)
**Blood test**	
White blood cell (10^9^/L), mean, SD	6.8 (3.7)
Platelet (10^9^/L), mean, SD	225.3 (90.4)
Leucocyte (%), mean, SD	22 (16)
**Comorbidities**	
Hypertension, n (%)	367 (32.6)
Diabetes mellitus, n (%)	204 (18.1)
Lipid disorder, n (%)	180 (16)
Coronary artery disease, n (%)	103 (9.2)
Liver dysfunction*, n (%)	65 (5.8)
Cancer, n (%)	65 (5.8)
AF/irregular rhythm*, n (%)	62 (5.5)
Bleeding diathesis*, n (%)	43 (3.8%)
Peripheral artery disease, n (%)	36 (3.2)
Heart failure, n (%)	32 (2.8)
Renal dysfunction*, n (%)	27 (2.4)
Hypertrophic cardiomyopathy, n (%)	23 (2.0)
Obstructive sleep apnea syndrome, n (%)	19 (1.7)
Hyperthyroidism, n (%)	4 (0.4)
**Surgery**, n (%)	33 (2.9)
Obstetrics and gynecology operation*, n (%)	22 (2.0)
General surgical operation*, n (%)	5 (0.4)
Orthopedic surgery*, n (%)	4 (0.4)
Neurosurgery*, n (%)	2 (0.2)
**Combined drugs**	
Calcium channel blockers, n (%)	227 (20.2)
Anti-acid drugs, n (%)	218 (19.4)
ϐ blockers, n (%)	172 (15.3)
Statins, n (%)	139 (12.4)
Insulin, n (%)	131 (11.6)
ACEI/ARB*, n (%)	96 (8.5)
Nitrates, n (%)	80 (7.1)
α-glucosidase inhibitor, n (%)	77 (6.8)
Biguanide, n (%)	68 (6.0)
Diuretics, n (%)	59 (5.2)
Spirolactone, n (%)	29 (2.6)
Sulfonylureas, n (%)	17 (1.5)
Digoxin, n (%)	10 (0.9)
Amiodarone, n (%)	3 (0.3)
**Anti-viral and related drugs**	
**Chinese herb**, n (%)	879 (78.1)
Antiviral drug*, n (%)	788 (70.0)
Arbidol, n (%)	703 (62.5)
Ribavirin, n (%)	195 (17.3)
Lopinavir/ritonavir, n (%)	65 (5.8)
Chloroquine phosphate, n (%)	43 (3.8)
Ganciclovir, n (%)	29 (2.6)
Remdesivir, n (%)	1 (0.1)
Immunomodulator*, n (%)	619 (55.0)
Antibiotics*, n (%)	583 (51.8)
**Glucocorticoid drugs**, n (%)	203 (18.0)

*For definitions of liver and renal dysfunction, lipid disorder, bleeding diathesis, see text.

Obstetrics and gynecology operations: midwifery, cesarean delivery, dilatation and curettage, oophorocystectomy. General surgical operations: appendectomy, interventional embolization of gastric varices, laparocolectomy. Orthopedic surgery: hip disarticulation, open reduction and internal fixation, hollow screw fixation. Neurosurgery: extraventricular drainage, minimally invasive drilling and drainage of subdural hematoma.

ACEI/ARB: angiotensin-converting enzyme inhibitor/angiotensin II receptor blocker.

Immunomodulator: interleukin-2, interferon, immunoglobulin, thymopentin.

Antibiotics: ceftizoxime sodium, ceftriaxone sodium, cefprozil, cefzon, ceftazidime, cefoperazone sodium sulbactam sodium, meropenem trihydrate, azithromycin, clarithromycin, levofloxacin, moxifloxacin, ornidazole, metronidazole, vancomycin, linezolid, teicoplanin.

There were 250 patients who received two antiviral drugs in sequence.

**Table 2:** Antithrombotic treatment in patients with COVID-2019.

	**Patients (n=1125)**
**Total Anticoagulants,** n (%)	**249 (22.1)**
**Oral anticoagulants,** n (%)	**87 (7.7)**
NOAC*, n (%)	82 (7.3)
Warfarin, n (%)	5 (0.4)
**Parenteral anticoagulants,** n (%)	**209 (18.6)**
LMWH*, n (%)	111 (9.9)
Heparin, n (%)	103 (9.2)
**Oral anticoagulants plus parenteral anticoagulants, n (%)**	**47 (4.2)**
**Total antiplatelets,** n (%)	**106 (9.4)**
Aspirin, n (%)	77 (6.8)
Clopidogrel, n (%)	49 (4.4)
Others, n (%)	1 (0.1)

* NOAC: non-vitamin K antagonist oral anticoagulant, rivaroxaban or dabigatran. LMWH: low molecular weight heparin. There were 21 patients with dual antiplatelet (aspirin plus clopidogrel); 5 patients who received both heparin and LMWH.

### Clinical outcomes

There were 82 (7.3%) thromboembolic events (deep vein thrombosis in 42 (3.7%), pulmonary embolism in 3 (0.3%), and ischemic stroke in 37 (3.3%) patients) and major bleeding events in 128 (11.4%) patients (Table 3). Approximately 25 (30%) patients with thromboembolism also suffered bleeding events (Table 3). All-cause death occurred in 91 (8.1%) patients.

**Table 3:** Thromboembolism and bleeding events during hospitalization.

	**Patients (n=1125)**
**Thromboembolism**, n (%)	82 (7.3)
Deep vein thromboembolism, n (%)	42 (3.7)
Ischaemic stroke, n (%)	37 (3.3)
Pulmonary thromboembolism, n (%)	3 (0.3)
Bleeding events*, n (%)	128 (11.4)
Gastrointestinal bleeds, n (%)	18 (1.6)
Intracranial haemorrhage, n (%)	6 (0.5)
Major bleeding*, n (%)	113 (10.0)
**Thromboembolism and bleeding events**, n (%)	25 (2.2)

* Major bleeding was defined as a drop of Hg ≥ 2g/L, or needed the infusion of RBC ≥ 2 unit. Bleeding events: included gastrointestinal bleeds, intracranial haemorrhage, and major bleeding.

Age was an independent risk factor for thromboembolism, bleeding events, and death, both individually and in combination (all p<0.05, Figure 1A–1E and Supplementary Tables 2, 3, 4).

**Figure 1A-B:**
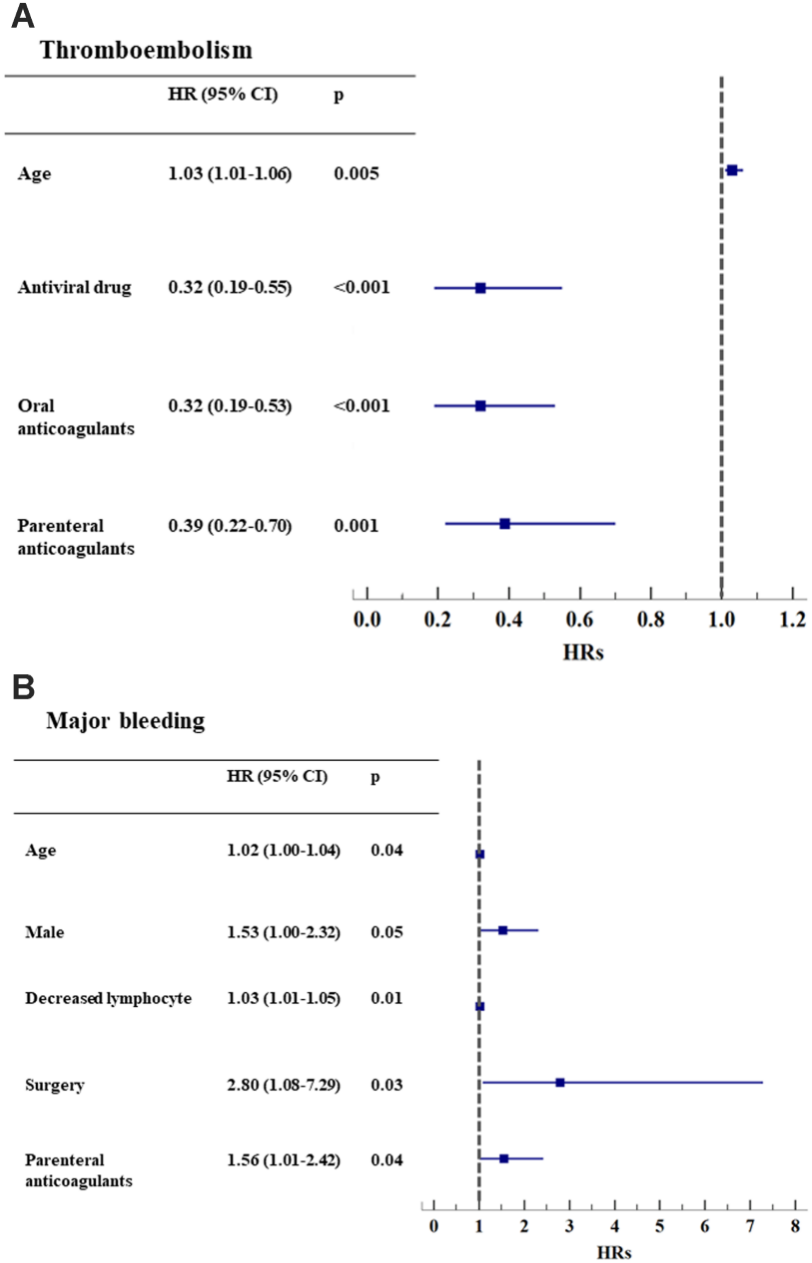
**Hazard ratios of clinical events, adjusting for baseline risk factors.** (**A**) Thromboembolism (n=82); (**B**) Major bleeding (n=113).

**Figure 1C-E:**
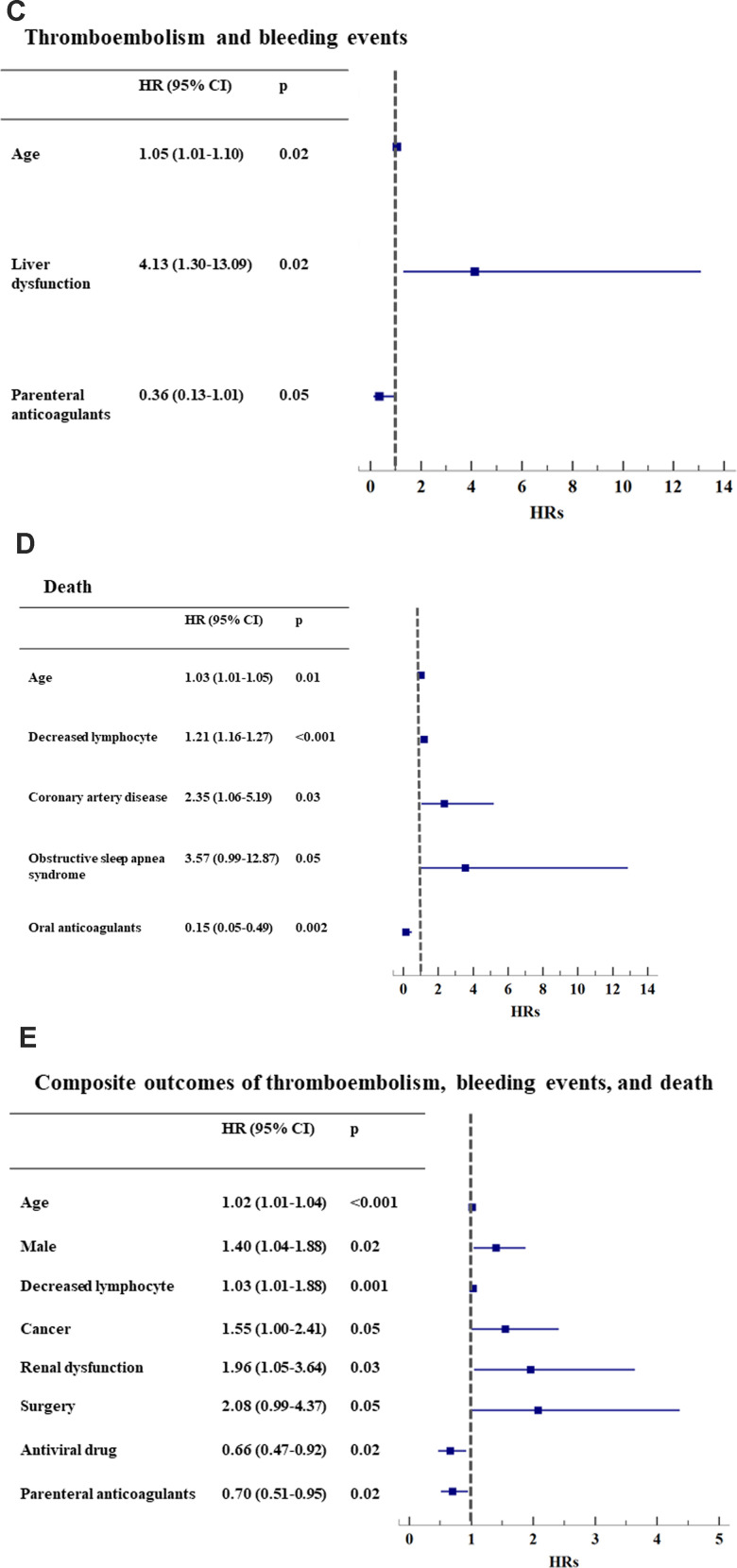
**Hazard ratios of clinical events, adjusting for baseline risk factors.** (**C**) Thromboembolism and bleeding events (n=25); (**D**) Death (n=91); (**E**) Composite outcomes of thromboembolism, bleeding events, and death (n=235). *HR: hazard ratio. CI: confidential interval.

In univariate analyses, hypertension, AF/irregular rhythm, heart failure, peripheral artery disease, cancer, obstructive sleep apnea syndrome, and renal dysfunction were associated with an increased risk for thromboembolism, whereas antiviral and antithrombotic treatment reduced the risk (all p <0.05, Figure 1A and Supplementary Table 2A).

After adjusting for the severity of COVID-19 infection, comorbidities, surgery, antiviral drugs, immunomodulators, Chinese herbs and antithrombotic drugs, the use of oral anticoagulants (HR 0.32, 0.19-0.53) and parenteral anticoagulants (HR 0.39, 0.22-0.70) reduced the risk for thromboembolism (all p<0.001, Figure 1A), as did antiviral drugs.

The presence of low lymphocyte counts (HR 1.03, 1.01-1.05, p=0.01) and surgery (HR 2.80, 1.08-7.29, p=0.03) independently predicted the risk for major bleeding, as did parenteral anticoagulants (HR 1.56, 1.01-2.42, p=0.04) (Figure 1B and Supplementary Table 2B).

Liver dysfunction (HR 4.13, 1.30-13.1, p=0.02) was an independent risk factor for patients who sustained both thromboembolism and bleeding events (Figure 1C and Supplementary Table 3). Oral anticoagulants reduced the risk for death (HR 0.15, 0.05-0.49, p=0.002, Figure 1D and Supplementary Table 2C).

After adjustment, parenteral anticoagulant use had a borderline effect on both thromboembolism and bleeding events (HR 0.36, 0.13-1.01, p=0.053, Figure 1C and Supplementary Table 3) and significantly reduced the risk for the composite outcome of thromboembolism, bleeding events and death (HR 0.70, 0.51-0.95, p=0.02, Figure 1E and Supplementary Table 2D). Other independent predictors of the composite outcome were age, male sex, low lymphocyte count, cancer, renal dysfunction and surgery (Figure 1E and Supplementary Table 2D).

### Subgroup analyses

**Figure 2:**
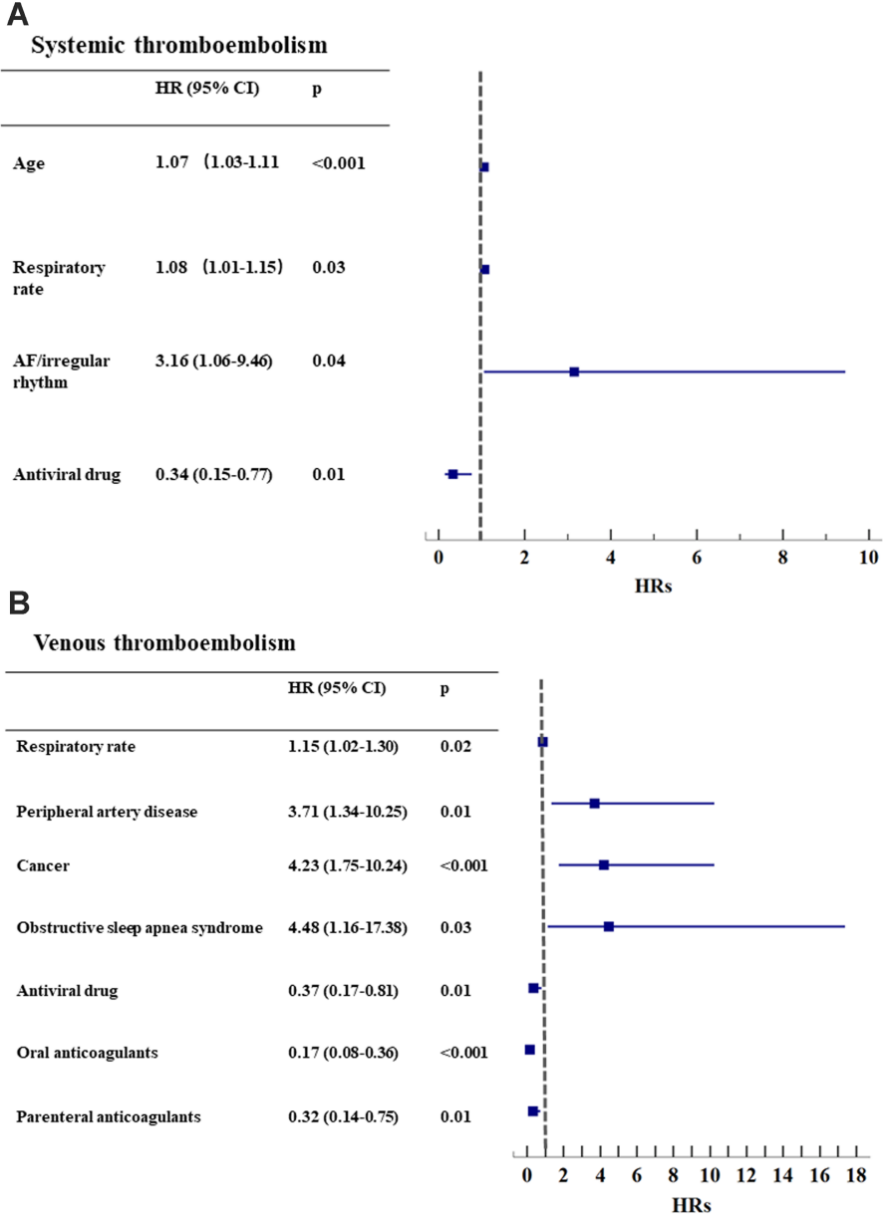
**Subgroup analysis for systemic and venous thromboembolism.** (**A**) Systemic thromboembolism (n=37); (**B**) Venous thromboembolism (n=45). * AF: atrial fibrillation. HR: hazard ratio. CI: confidential interval.

The subgroups of patients with systemic and venous thromboembolism were analyzed separately. In the multivariate analysis, age (HR 1.07, 1.03-1.11, p<0.001), AF rhythm (HR 3.16, 1.06-9.46, p=0.04) and increased respiratory rate (HR 1.08, 1.01-1.15, p=0.025) were independent risk factors for systemic thromboembolism, but antiviral drugs reduced this risk (HR 0.34, 0.15-0.77, p=0.01, Figure 2A and Supplementary Table 4A). Of these patients with systemic thromboembolism, only 5 (13.5%) received oral anticoagulants, and 16 (43.2%) received parenteral anticoagulants (Supplementary Table 5).

The risk of venous thromboembolism was increased with peripheral artery disease, cancer, obstructive sleep apnea syndrome and increased respiratory rate (all p<0.05) but was reduced by the use of antiviral drugs, oral anticoagulants and parenteral anticoagulants (all p<0.01, Figure 2B and Supplementary Table 4B). There were 25 (55.6%) patients on OACs and 33 (73.3%) taking parenteral anticoagulants (Supplementary Table 5).

## DISCUSSION

In this series of COVID-19 patients, our principal findings are as follows: i) thromboembolic, major bleeding and mortality risks were high, with crude in-hospital rates of 7.3%, 11.4% and 8.1%, respectively; ii) age was an independent risk factor for all clinical events, liver dysfunction predicted both thromboembolism and bleeding events, whereas low lymphocyte and surgery were associated with major bleeding; iii) both oral and parenteral anticoagulants reduced the risk for thromboembolism; specifically, parenteral anticoagulants reduced the risk for the composite outcome of thromboembolism, major bleeding and death; and iv) subgroup analysis showed that AF rhythm increased the risk for systemic thromboembolism.

In several small studies, the incidences of venous thromboembolism (deep vein thrombosis, pulmonary embolism) were reported in 25% to 31% of critically ill COVID-19 patients in the intensive care unit [8, 9]. Excessive inflammation has been suggested as one of the main drivers of blood coagulation activation in severely ill patients [10]. In addition to the COVID-19 infection itself, several comorbidities, such as hypertension, AF/irregular rhythm, heart failure, peripheral artery disease, cancer, obstructive sleep apnea syndrome and renal dysfunction, may contribute to thromboembolic risk, even in mild or moderate COVID-19 patients. Ischemic stroke was also common, second to venous thromboembolism, suggesting that any thromboprophylaxis approach should aim to prevent both systemic and venous thromboembolic events, even for mild or moderately ill COVID-19 patients.

We are unaware of reported major bleeding outcomes in large cohorts of patients with COVID-19. In the present study, we observed that the rate of bleeding events was 11.4%, which was associated with the severity of COVID-19 itself and any surgery/invasive procedure. Multiorgan dysfunction could be caused by severe COVID-19 infection, and liver dysfunction was independently associated with both thromboembolic and bleeding risks. Impaired liver synthetic function of coagulation factors has been reported in patients with COVID-19 [13].

The present study demonstrates that the use of both oral (94% on nonvitamin K antagonist oral anticoagulants) and parenteral anticoagulants was associated with reduced thromboembolism over a mean follow-up of 21 days as well as a reduced risk for death. When taking the composite outcome of thromboembolism, major bleeding and death into consideration, the use of parenteral anticoagulants showed a significant beneficial effect.

In addition, subgroup analyses demonstrated that AF rhythm independently increased the risk for systemic thromboembolism by threefold. There are reports that 17%-44% of COVID-19 patients suffer from cardiac arrhythmias (commonly AF) [14]. Indeed, inflammation initiates and perpetuates AF and AF-related thrombosis [15]. Our study emphasizes that in patients with COVID-19, detection and awareness of AF and anticoagulation should be considered.

### Limitations

There were several limitations in this study. First, the low rates of confirmed thromboembolism may reflect medical resource limitations, for example, CT scans, especially in the early stages of the COVID-19 outbreak in China. Second, we used electronic medical records in the present study, but the details depended on the thoroughness of the data collection of the risk factors and clinical events. Finally, neither the dosages and duration of LMWH nor the doses of NOACs were formally evaluated.

## CONCLUSIONS

Patients with COVID-19 were at high risk for thromboembolic and bleeding events as well as mortality. Anticoagulant use, especially parenteral anticoagulants, significantly reduced the risk for composite outcomes of thromboembolism, bleeding events and death. The presence of AF was a contributor to systemic thromboembolism in COVID-19 patients.

## MATERIALS AND METHODS

Patients with COVID-19 admitted to Union Hospital, Wuhan, Hubei, China between January 08, 2020 and April 7, 2020 were included in the study. For this study, we used the electronic medical records database, which recorded the patient’s medical history, therapeutic procedure(s), and laboratory data, which were related to clinical outcomes and mortality. All the data were first collected and by a group of physicians, then data were independently reviewed and validated by a group of physicians. The medical ethics committee of Tongji Medical College, Huazhong University of Science and Technology approved the present study (Approval No. 2020-S068).

### Study population

The inclusion criteria were patients with a confirmed diagnosis of COVID-19. The diagnosis of COVID-19 was made according to the Guidance for Coronavirus Disease 2019 (6th edition) released by the National Health Commission of China [16] and confirmed by clinical medical history, nucleic acid testing, and CT scan. Rates of thromboembolism, major bleeding events, and all-cause death were recorded. Exclusion criteria included patients with incomplete medical records, therapeutic procedures, or laboratory data. Over the study period, a total of 1,132 patients with confirmed COVID-19 were selected, and after excluding 7 patients with incomplete medical records, we identified 1,125 patients for the final study analysis. We recorded data on the severity of COVID-19 infection, comorbidities, surgery, drug therapies, and use of antithrombotic drugs. Decreased lymphocyte count and respiratory rate were taken as the indexes of the severity of COVID-19 infection [2, 17–19]. Patient comorbidities, including hypertension, diabetes mellitus, lipid disorders, coronary artery disease, cancer, peripheral artery disease, heart failure, hypertrophic cardiomyopathy, obstructive sleep apnea syndrome, and hyperthyroidism, etc. were collected from electronic medical records based on the diagnostic criteria of their retrospective guidelines. Atrial fibrillation (AF) was defined as the diagnosis of AF irregular rhythm in the physical examination recorded in electronic medical records. Liver dysfunction was defined by serum alanine aminotransferase, aspartate transaminase, or alkaline phosphatase levels > 3x the upper limit of normal or known liver disease. Renal dysfunction was defined by serum creatinine ≥ 200 μmol/l (2.26 mg/dL). Lipid disorder was defined as fasting total cholesterol > 240 mg/dl (> 6.2 mmol/l) or low-density lipoprotein cholesterol > 160 mg/dl (> 4.1 mmol/l) or treatment with any lipid-lowering drug. Bleeding diathesis was defined as hemoglobin (Hg) < 80 g/L or platelet (PLT) count < 50x10^9/L on admission. Information on surgery was collected from inpatient medical records. The use of antiviral drugs, antibiotics, immunomodulators, glucocorticoid drugs, and Chinese herbs were recorded, as were oral anticoagulants, parenteral anticoagulants (heparin, LMWH), and antiplatelet drugs.

### Outcomes

Thromboembolism, major bleeding events, and death in the hospital were taken as the outcomes. Thromboembolism included systemic (ischemic stroke) and venous thromboembolism (deep vein thromboembolism and pulmonary embolism). Major bleeding events included intracranial and extracranial hemorrhage, a drop in Hb ≥ 2 g/L, or the requirement of red blood cell transfusion ≥ 2 units. All clinical events were diagnosed by hospital specialists and recorded in the electronic medical records.

### Statistical analysis

Continuous variables were tested for normality by the Kolmogorov-Smirnov test. Those with a normal distribution are presented as the mean (standard deviation, SD). The rates of thromboembolism, major bleeding events, and deaths were calculated.

Univariate analysis was used to analyze underlying risk factors associated with thromboembolism, major bleeding, and death. Multivariable Cox proportional hazards models were used to analyze risk factors associated with clinical events after adjusting for underlying risk factors. We adjusted thromboembolic and bleeding outcomes for potential confounders, including the severity of COVID-19 (decreased lymphocyte count and respiratory rate), related drugs (antiviral drugs, immunomodulators, and Chinese herbs), comorbidities, surgery, and antithrombotic drugs (oral anticoagulants, parenteral anticoagulants, and antiplatelets) (Supplementary Figure 1).

Independent risk factors for the composite of “thromboembolism and bleeding” and the composite outcome of “thromboembolism, bleeding events, and death” were determined. Hazard ratios (HRs, i.e., hazard of intervention to control) and 95% confidence intervals (CIs) were estimated. Next, the impact of anticoagulants (oral and parenteral) on thromboembolism, major bleeding, and death was studied using Cox proportional hazards models after adjustment for 19 clinical factors: age, male sex, decreased lymphocyte count, respiratory rate, hypertension, coronary artery disease, AF/irregular rhythm, heart failure, diabetes mellitus, peripheral artery disease, cancer, obstructive sleep apnea syndrome, liver dysfunction, renal dysfunction, surgical procedures, and drug therapies (antiviral drugs, immunomodulators, Chinese herbs, and antiplatelet drugs).

Subgroup analysis was carried out for systemic and venous thromboembolism. A two-sided p-value < 0.05 was considered statistically significant. The statistical analysis was performed using IBM SPSS Statistics, version 22.0 (SPSS Inc).

## Supplementary Material

Supplementary Figure 1

Supplementary Tables
