# Supplementary material for: Risk factors for systemic and venous thromboembolism, mortality and bleeding risks in 1125 patients with COVID-19: relationship with anticoagulation status
Source: Aging (Albany NY). 2021 Mar 26;13(7):9225–42. doi: 10.18632/aging.202769 (PMC8064159; doi:10.18632/aging.202769)
Supplement: Supplementary Figure 1 [file aging-13-202769-s001.pdf]

SUPPLEMENTARY FIGURE

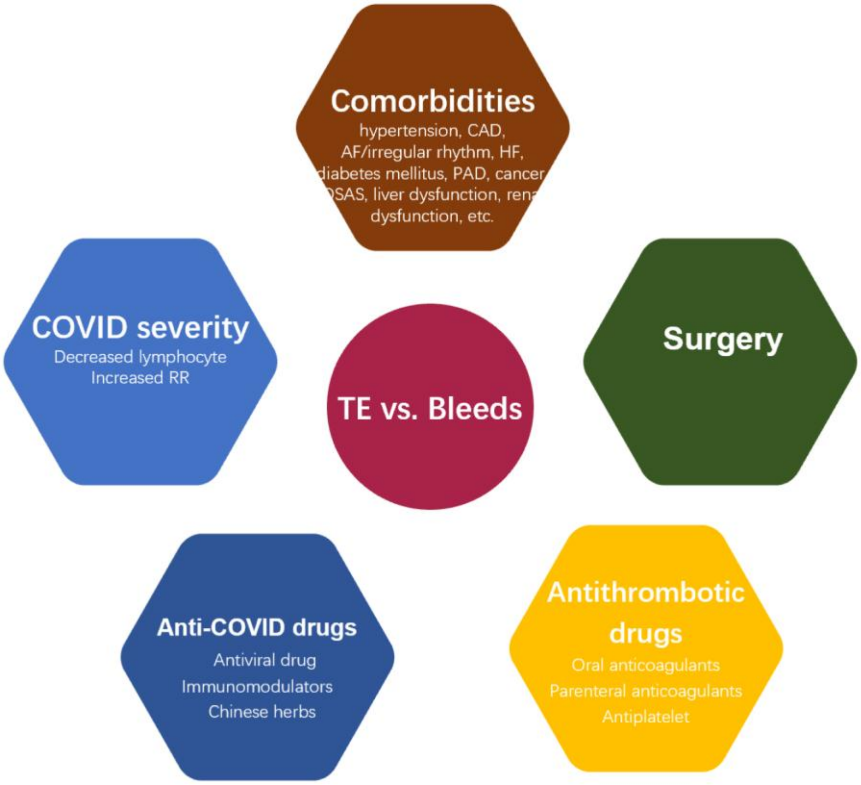

Supplementary Figure 1. The underlying factors in analysis for thromboembolic and bleeding risk in patients with COVID-2019. CAD: coronary artery disease. HF: heart failure. PAD: peripheral artery disease. OSAS: Obstructive sleep apnea syndrome. RR: respiratory rate.
