# Supplementary material for: Risk factors for systemic and venous thromboembolism, mortality and bleeding risks in 1125 patients with COVID-19: relationship with anticoagulation status
Source: Aging (Albany NY). 2021 Mar 26;13(7):9225–42. doi: 10.18632/aging.202769 (PMC8064159; doi:10.18632/aging.202769)
Supplement: Supplementary Tables [file aging-13-202769-s002.pdf]

## SUPPLEMENTARY TABLES

**Supplementary Table 1. Specialties of hospitalised patients when first admitted.**

| | Sites (n=1125) | Rate, (%) |
|---|---|---|
| Surgery | 491 | 43.6% |
| Obstetrics & Gynaecology | 141 | 12.5% |
| Cardiology | 91 | 8.1% |
| Ear/Nose/Throat | 90 | 8.0% |
| Respiratory | 74 | 6.6% |
| Haematology | 71 | 6.3% |
| Neurology | 57 | 5.1% |
| Ophthalmology | 54 | 4.8% |
| Gastroenterology | 31 | 2.8% |
| Intensive care unit | 1 | 0.1% |
| Others | 24 | 2.1% |

**Supplementary Table 2. Hazard ratios for clinical events.**

**A. Thromboembolism (n=82).**

| | Univariate analysis | | | | Multivariate analysis | | | |
|---|---|---|---|---|---|---|---|---|
| | HR | 95% CI | | P | HR | 95% CI | | P |
| | | Lower limit | Higher limit | | | Lower limit | Higher limit | |
| Age | 1.06 | 1.04 | 1.08 | **<0.001** | 1.03 | 1.01 | 1.06 | **0.005** |
| Male | 0.85 | 0.55 | 1.32 | 0.46 | 0.88 | 0.53 | 1.48 | 0.64 |
| Decreased lymphocyte | 1.00 | 0.98 | 1.01 | 0.81 | 1.01 | 1.00 | 1.01 | 0.25 |
| Respiratory rate | 0.98 | 0.92 | 1.04 | 0.47 | 1.01 | 0.95 | 1.08 | 0.78 |
| Hypertension | 2.86 | 1.82 | 4.48 | **<0.001** | 1.62 | 0.95 | 2.77 | 0.08 |
| Coronary artery disease | 1.47 | 0.79 | 2.74 | 0.22 | 0.51 | 0.21 | 1.21 | 0.12 |
| AF/irregular rhythm | 2.87 | 1.51 | 5.43 | **0.001** | 1.90 | 0.85 | 4.27 | 0.12 |
| Heart failure | 3.67 | 1.76 | 7.66 | **0.001** | 1.92 | 0.73 | 5.03 | 0.18 |
| Diabetes mellitus | 0.96 | 0.55 | 1.67 | 0.89 | 0.56 | 0.27 | 1.13 | 0.11 |
| Peripheral artery disease | 2.90 | 1.49 | 5.67 | **0.002** | 2.10 | 0.92 | 4.80 | 0.08 |
| Cancer | 2.84 | 1.54 | 5.26 | **0.001** | 1.94 | 0.94 | 4.02 | 0.07 |
| Obstructive sleep apnea syndrome | 3.75 | 1.37 | 10.28 | **0.01** | 2.06 | 0.69 | 6.11 | 0.19 |
| Liver dysfunction | 1.58 | 0.73 | 3.43 | 0.25 | 1.41 | 0.55 | 3.61 | 0.47 |
| Renal dysfunction | 2.88 | 1.05 | 7.90 | **0.04** | 1.73 | 0.54 | 5.56 | 0.35 |
| Surgery | 1.82 | 0.57 | 5.79 | 0.31 | 2.17 | 0.60 | 7.88 | 0.24 |
| Antiviral drug | 0.28 | 0.18 | 0.44 | **<0.001** | 0.32 | 0.19 | 0.55 | **<0.001** |
| Immunomodulator | 0.47 | 0.30 | 0.73 | **0.001** | 0.62 | 0.37 | 1.04 | 0.07 |
| Chinese herbs | 1.39 | 0.85 | 2.26 | 0.19 | 1.22 | 0.68 | 2.19 | 0.52 |
| Oral anticoagulants | 0.24 | 0.15 | 0.38 | **<0.001** | 0.32 | 0.19 | 0.53 | **<0.001** |
| Parenteral anticoagulants | 0.23 | 0.14 | 0.37 | **<0.001** | 0.39 | 0.22 | 0.70 | **0.001** |
| Antiplatelet | 0.45 | 0.27 | 0.75 | **0.002** | 1.63 | 0.80 | 3.31 | 0.18 |

* AF: atrial fibrillation. HR: hazard ratio. CI: confidence interval.

**B. Major bleeding (n=113).**

| | Univariate analysis | | | | Multivariate analysis | | | |
|---|---|---|---|---|---|---|---|---|
| | HR | 95% CI | | P | HR | 95% CI | | P |
| | | Lower limit | Higher limit | | | Lower limit | Higher limit | |
| Age | 1.03 | 1.01 | 1.04 | **<0.001** | 1.02 | 1.00 | 1.04 | **0.04** |
| Male | 1.50 | 1.03 | 2.19 | **0.03** | 1.53 | 1.00 | 2.32 | **0.05** |
| Decreased lymphocyte | 1.05 | 1.03 | 1.07 | **<0.001** | 1.03 | 1.01 | 1.05 | **0.01** |
| Respiratory rate | 1.02 | 0.97 | 1.06 | 0.46 | 1.00 | 0.96 | 1.05 | 0.82 |
| Hypertension | 1.47 | 1.01 | 2.14 | **0.05** | 1.22 | 0.80 | 1.87 | 0.35 |
| Coronary artery disease | 1.13 | 0.63 | 2.01 | 0.69 | 1.24 | 0.61 | 2.51 | 0.55 |
| AF/irregular rhythm | 0.46 | 0.15 | 1.45 | 0.19 | 0.47 | 0.14 | 1.54 | 0.21 |
| Heart failure | 1.32 | 0.52 | 3.33 | 0.56 | 1.11 | 0.42 | 2.94 | 0.83 |
| Diabetes mellitus | 1.01 | 0.64 | 1.60 | 0.98 | 1.00 | 0.60 | 1.65 | 0.99 |
| Peripheral artery disease | 0.98 | 0.40 | 2.40 | 0.96 | 1.06 | 0.38 | 2.96 | 0.92 |
| Cancer | 1.62 | 0.84 | 3.10 | 0.15 | 1.17 | 0.58 | 2.37 | 0.65 |
| Obstructive sleep apnea syndrome | 1.90 | 0.60 | 5.98 | 0.28 | 1.68 | 0.51 | 5.50 | 0.39 |
| Liver dysfunction | 1.88 | 1.00 | 3.50 | **0.05** | 1.74 | 0.88 | 3.45 | 0.11 |
| Renal dysfunction | 3.12 | 1.37 | 7.12 | **0.01** | 1.42 | 0.50 | 4.00 | 0.51 |
| Surgery | 2.79 | 1.22 | 6.37 | **0.02** | 2.80 | 1.08 | 7.29 | **0.03** |
| Antiviral drug | 1.12 | 0.68 | 1.85 | 0.66 | 1.07 | 0.61 | 1.85 | 0.82 |
| Immunomodulator | 1.32 | 0.89 | 1.96 | 0.17 | 1.18 | 0.77 | 1.82 | 0.45 |
| Chinese herbs | 1.25 | 0.84 | 1.87 | 0.28 | 1.22 | 0.78 | 1.89 | 0.39 |
| Oral anticoagulants | 1.42 | 0.88 | 2.29 | 0.16 | 1.15 | 0.69 | 1.93 | 0.59 |
| Parenteral anticoagulants | 2.38 | 1.63 | 3.48 | **<0.001** | 1.56 | 1.01 | 2.42 | **0.04** |
| Antiplatelet | 0.75 | 0.40 | 1.40 | 0.37 | 0.52 | 0.25 | 1.08 | 0.08 |

* AF: atrial fibrillation. HR: hazard ratio. CI: confidence interval.

**C. Death (n=91).**

| | Univariate analysis | | | | Multivariate analysis | | | |
|---|---|---|---|---|---|---|---|---|
| | HR | 95% CI | | P | HR | 95% CI | | P |
| | | Lower limit | Higher limit | | | Lower limit | Higher limit | |
| Age | 1.05 | 1.04 | 1.07 | **<0.001** | 1.03 | 1.01 | 1.05 | **0.01** |
| Male | 1.68 | 1.10 | 2.57 | **0.02** | 1.16 | 0.69 | 1.97 | 0.57 |
| Decreased lymphocyte | 1.21 | 1.16 | 1.26 | **<0.001** | 1.21 | 1.16 | 1.27 | **<0.001** |
| Respiratory rate | 1.04 | 1.00 | 1.09 | **0.04** | 1.00 | 0.96 | 1.04 | 0.96 |
| Hypertension | 1.54 | 1.01 | 2.33 | **0.04** | 1.00 | 0.59 | 1.69 | 0.99 |
| Coronary artery disease | 1.74 | 0.98 | 3.07 | 0.06 | 2.35 | 1.06 | 5.19 | **0.03** |
| AF/irregular rhythm | 0.76 | 0.28 | 2.07 | 0.59 | 0.73 | 0.19 | 2.75 | 0.64 |
| Heart failure | 3.09 | 1.43 | 6.68 | **0.004** | 1.28 | 0.45 | 3.63 | 0.65 |
| Diabetes mellitus | 1.85 | 1.18 | 2.90 | **0.01** | 1.26 | 0.72 | 2.20 | 0.43 |
| Peripheral artery disease | 0.87 | 0.28 | 2.77 | 0.82 | 0.76 | 0.18 | 3.25 | 0.71 |
| Cancer | 1.81 | 0.91 | 3.60 | 0.09 | 1.43 | 0.67 | 3.07 | 0.36 |
| Obstructive sleep apnea syndrome | 2.06 | 0.65 | 6.52 | 0.22 | 3.57 | 0.99 | 12.87 | **0.05** |
| Liver dysfunction | 2.50 | 1.36 | 4.59 | **0.003** | 1.45 | 0.70 | 3.01 | 0.32 |
| Renal dysfunction | 6.40 | 3.40 | 12.01 | **<0.001** | 1.74 | 0.75 | 4.05 | 0.20 |
| Surgery | 0.40 | 0.06 | 2.84 | 0.36 | 0.91 | 0.12 | 6.98 | 0.93 |
| Antiviral drug | 1.11 | 0.68 | 1.82 | 0.67 | 1.76 | 0.89 | 3.51 | 0.11 |
| Immunomodulator | 1.32 | 0.86 | 2.02 | 0.21 | 1.00 | 0.57 | 1.74 | 1.00 |

| | | | | | | | | |
|---|---|---|---|---|---|---|---|---|
| Chinese herbs | 1.43 | 0.91 | 2.23 | 0.12 | 0.92 | 0.54 | 1.57 | 0.76 |
| Oral anticoagulants | 0.31 | 0.10 | 0.98 | **0.05** | 0.15 | 0.05 | 0.49 | **0.002** |
| Parenteral anticoagulants | 0.60 | 0.32 | 0.77 | **0.002** | 0.82 | 0.48 | 1.39 | 0.47 |
| Antiplatelet | 0.59 | 0.26 | 1.35 | 0.21 | 0.49 | 0.18 | 1.32 | 0.16 |

* AF: atrial fibrillation. HR: hazard ratio. CI: confidence interval.

**D. Composite outcomes of thromboembolism, bleeding events, and death (n=235).**

| | Univariate analysis | | | | Multivariate analysis | | | |
|---|---|---|---|---|---|---|---|---|
| | HR | 95% CI | | P | HR | 95% CI | | P |
| | | Lower limit | Higher limit | | | Lower limit | Higher limit | |
| Age | 1.04 | 1.03 | 1.05 | **<0.001** | 1.02 | 1.01 | 1.04 | **<0.001** |
| Male | 1.33 | 1.03 | 1.73 | **0.03** | 1.40 | 1.04 | 1.88 | **0.02** |
| Decreased lymphocyte | 1.05 | 1.03 | 1.06 | **<0.001** | 1.03 | 1.01 | 1.05 | **0.001** |
| Respiratory rate | 1.01 | 0.98 | 1.04 | 0.38 | 1.01 | 0.98 | 1.04 | 0.60 |
| Hypertension | 1.72 | 1.33 | 2.23 | **<0.001** | 1.23 | 0.91 | 1.66 | 0.18 |
| Coronary artery disease | 1.23 | 0.83 | 1.81 | 0.31 | 0.99 | 0.61 | 1.62 | 0.97 |
| AF/irregular rhythm | 1.04 | 0.59 | 1.81 | 0.90 | 0.93 | 0.50 | 1.72 | 0.81 |
| Heart failure | 2.10 | 1.23 | 3.58 | **0.01** | 1.32 | 0.71 | 2.45 | 0.38 |
| Diabetes mellitus | 1.15 | 0.85 | 1.57 | 0.36 | 0.94 | 0.66 | 1.34 | 0.75 |
| Peripheral artery disease | 1.27 | 0.72 | 2.23 | 0.40 | 1.15 | 0.61 | 2.17 | 0.66 |
| Cancer | 2.18 | 1.47 | 3.24 | **<0.001** | 1.55 | 1.00 | 2.41 | **0.05** |
| Obstructive sleep apnea syndrome | 2.14 | 1.01 | 4.54 | **0.05** | 1.55 | 0.71 | 3.38 | 0.27 |
| Liver dysfunction | 1.69 | 1.09 | 2.63 | **0.02** | 1.57 | 0.96 | 2.56 | 0.07 |
| Renal dysfunction | 3.56 | 2.11 | 6.01 | **<0.001** | 1.96 | 1.05 | 3.64 | **0.03** |
| Surgery | 1.74 | 0.89 | 3.40 | 0.10 | 2.08 | 0.99 | 4.37 | **0.05** |
| Antiviral drug | 0.59 | 0.44 | 0.78 | **<0.001** | 0.66 | 0.47 | 0.92 | **0.02** |
| Immunomodulator | 0.81 | 0.62 | 1.05 | 0.11 | 0.89 | 0.66 | 1.20 | 0.44 |
| Chinese herbs | 1.19 | 0.91 | 1.57 | 0.21 | 1.06 | 0.78 | 1.44 | 0.72 |
| Oral anticoagulants | 0.64 | 0.46 | 0.89 | **0.01** | 0.79 | 0.55 | 1.13 | 0.20 |
| Parenteral anticoagulants | 0.47 | 0.36 | 0.61 | **<0.001** | 0.70 | 0.51 | 0.95 | **0.02** |
| Antiplatelet | 1.01 | 0.68 | 1.51 | 0.95 | 0.68 | 0.41 | 1.11 | 0.12 |

* AF: atrial fibrillation. HR: hazard ratio. CI: confidence interval.

**Supplementary Table 3. Hazard ratios for composite of thromboembolism and bleeding events (n=25).**

| | Hazard ratios | Confidence interval | | P |
|---|---|---|---|---|
| | | Low | High | |
| Age | 1.05 | 1.01 | 1.10 | **0.02** |
| Male | 1.14 | 0.46 | 2.78 | 0.78 |
| Decreased lymphocyte | 0.98 | 0.94 | 1.03 | 0.47 |
| Respiratory rate | 0.91 | 0.79 | 1.05 | 0.18 |
| Hypertension | 1.17 | 0.46 | 2.97 | 0.75 |
| Coronary artery disease | 0.43 | 0.09 | 2.13 | 0.30 |
| AF/irregular rhythm | 1.23 | 0.26 | 5.74 | 0.79 |
| Heart failure | 2.29 | 0.48 | 10.85 | 0.30 |
| Diabetes mellitus | 0.66 | 0.20 | 2.20 | 0.50 |
| Peripheral artery disease | 1.91 | 0.41 | 8.80 | 0.41 |
| Cancer | 0.85 | 0.18 | 4.11 | 0.84 |
| Obstructive sleep apnea syndrome | 3.63 | 0.67 | 19.53 | 0.13 |
| Liver dysfunction | 4.13 | 1.30 | 13.09 | **0.02** |
| Renal dysfunction | 2.34 | 0.36 | 15.27 | 0.37 |
| Antiviral drug | 0.46 | 0.16 | 1.38 | 0.17 |
| Immunomodulator | 1.03 | 0.39 | 2.71 | 0.95 |
| Chinese herbs | 2.06 | 0.62 | 6.84 | 0.24 |
| Oral anticoagulants | 1.84 | 0.71 | 4.78 | 0.21 |
| Parenteral anticoagulants | 0.36 | 0.13 | 1.01 | **0.05** |
| Antiplatelet | 1.21 | 0.36 | 4.04 | 0.76 |

* AF: atrial fibrillation.

**Supplementary Table 4. Subgroup analysis.**

**A. Systemic thromboembolism (n=37).**

| | Hazard ratios | Confidence interval | | P |
|---|---|---|---|---|
| | | Low | High | |
| Age | 1.07 | 1.03 | 1.11 | **<0.001** |
| Male | 0.89 | 0.41 | 1.95 | 0.77 |
| Decreased lymphocyte | 1.00 | 0.96 | 1.04 | 0.87 |
| Respiratory rate | 1.08 | 1.01 | 1.15 | **0.03** |
| Hypertension | 1.97 | 0.89 | 4.33 | 0.09 |
| Coronary artery disease | 0.39 | 0.10 | 1.48 | 0.17 |
| AF/irregular rhythm | 3.16 | 1.06 | 9.46 | **0.04** |
| Heart failure | 0.94 | 0.15 | 5.93 | 0.94 |
| Diabetes mellitus | 0.73 | 0.25 | 2.07 | 0.55 |
| Peripheral artery disease | 1.30 | 0.27 | 6.32 | 0.75 |
| Cancer | 0.24 | 0.03 | 1.91 | 0.18 |
| Obstructive sleep apnea syndrome | 2.86 | 0.61 | 13.42 | 0.18 |
| Liver dysfunction | 0.42 | 0.04 | 3.87 | 0.44 |
| Renal dysfunction | 1.63 | 0.17 | 15.69 | 0.67 |
| Surgery | 3.47 | 0.44 | 27.41 | 0.24 |
| Antiviral drug | 0.34 | 0.15 | 0.77 | **0.01** |
| Immunomodulator | 0.64 | 0.28 | 1.47 | 0.30 |
| Chinese herbs | 1.01 | 0.44 | 2.31 | 0.98 |
| Oral anticoagulants | 0.61 | 0.20 | 1.86 | 0.39 |
| Parenteral anticoagulants | 0.47 | 0.21 | 1.08 | 0.08 |
| Antiplatelet | 1.51 | 0.53 | 4.30 | 0.44 |

* AF: atrial fibrillation.

**B. Venous thromboembolism (n=45).**

| | Hazard ratios | Confidence interval | | P |
|---|---|---|---|---|
| | | Low | High | |
| Age | 1.02 | 0.99 | 1.05 | 0.14 |
| Male | 1.13 | 0.54 | 2.36 | 0.74 |
| Decreased lymphocyte | 1.00 | 0.98 | 1.01 | 0.64 |
| Respiratory rate | 1.15 | 1.02 | 1.30 | **0.02** |
| Hypertension | 1.29 | 0.60 | 2.78 | 0.51 |
| Coronary artery disease | 0.86 | 0.26 | 2.85 | 0.80 |
| AF/irregular rhythm | 1.39 | 0.42 | 4.59 | 0.59 |
| Heart failure | 2.73 | 0.79 | 9.49 | 0.11 |
| Diabetes mellitus | 0.45 | 0.16 | 1.30 | 0.14 |
| Peripheral artery disease | 3.71 | 1.34 | 10.25 | **0.01** |
| Cancer | 4.23 | 1.75 | 10.24 | **<0.001** |
| Obstructive sleep apnea syndrome | 4.48 | 1.16 | 17.38 | **0.03** |
| Liver dysfunction | 1.69 | 0.47 | 6.12 | 0.42 |
| Renal dysfunction | 1.55 | 0.38 | 6.25 | 0.54 |
| Surgery | 2.16 | 0.39 | 11.94 | 0.38 |
| Antiviral drug | 0.37 | 0.17 | 0.81 | **0.01** |
| Immunomodulator | 0.57 | 0.28 | 1.19 | 0.14 |
| Chinese herbs | 0.96 | 0.41 | 2.25 | 0.92 |

| | | | | |
|---|---|---|---|---|
| Oral anticoagulants | 0.17 | 0.08 | 0.36 | **<0.001** |
| Parenteral anticoagulants | 0.32 | 0.14 | 0.75 | **0.01** |
| Antiplatelet | 1.45 | 0.50 | 4.20 | 0.50 |

* AF: atrial fibrillation.

**Supplementary Table 5. Antithrombotic treatment in patients with venous thromboembolism and systemic thromboembolism.**

| | Patients with systemic thromboembolism (n=37) | Patients with venous thromboembolism (n=45) |
|---|---|---|
| Oral anticoagulants, n (%) | 5 (13.5) | 25 (55.6) |
| Parenteral anticoagulants, n (%) | 16 (43.2) | 33 (73.3) |
| Antiplatelet, n (%) | 9 (24.3) | 9 (20.0) |
